# From GWAS to Function: Using Functional Genomics to Identify the Mechanisms Underlying Complex Diseases

**DOI:** 10.3389/fgene.2020.00424

**Published:** 2020-05-13

**Authors:** Eddie Cano-Gamez, Gosia Trynka

**Affiliations:** ^1^Wellcome Sanger Institute, Wellcome Genome Campus, Hinxton, United Kingdom; ^2^Open Targets, Wellcome Genome Campus, Cambridge, United Kingdom

**Keywords:** GWAS, SNP enrichment, colocalization analysis, TWAS, single-cell RNA seq, eQTL, QTL

## Abstract

Genome-wide association studies (GWAS) have successfully mapped thousands of loci associated with complex traits. These associations could reveal the molecular mechanisms altered in common complex diseases and result in the identification of novel drug targets. However, GWAS have also left a number of outstanding questions. In particular, the majority of disease-associated loci lie in non-coding regions of the genome and, even though they are thought to play a role in gene expression regulation, it is unclear which genes they regulate and in which cell types or physiological contexts this regulation occurs. This has hindered the translation of GWAS findings into clinical interventions. In this review we summarize how these challenges have been addressed over the last decade, with a particular focus on the integration of GWAS results with functional genomics datasets. Firstly, we investigate how the tissues and cell types involved in diseases can be identified using methods that test for enrichment of GWAS variants in genomic annotations. Secondly, we explore how to find the genes regulated by GWAS loci using methods that test for colocalization of GWAS signals with molecular phenotypes such as quantitative trait loci (QTLs). Finally, we highlight potential future research avenues such as integrating GWAS results with single-cell sequencing read-outs, designing functionally informed polygenic risk scores (PRS), and validating disease associated genes using genetic engineering. These tools will be crucial to identify new drug targets for common complex diseases.

## Introduction

Common non-communicable diseases such as autoimmunities, neurodegeneration, and cardiovascular disease are among the most pressing challenges in present day healthcare. These conditions are influenced by the interaction between a genetic predisposition and environmental or lifestyle factors (Smith et al., 2005). As opposed to rare diseases, which are often caused by the dysfunction of a single gene, common diseases are complex traits, i.e., they are influenced by the added contribution of thousands of common genetic variants, each having a small individual effect on the phenotype (Hindorff et al., 2011). This makes studying complex diseases challenging, as their genetic architecture follows a polygenic rather than a Mendelian model (Visscher and Goddard, 2019).

Genome-wide association studies (GWAS) are designed to map the polygenic architecture of common diseases by identifying genetic variants present at a significantly higher frequency in individuals with disease than in the healthy population (Wellcome Trust Case Control Consortium, 2007). Over the last 12 years, GWAS have grown significantly both in sample size and in the number of investigated traits (Visscher et al., 2017), with 128,550 associations and over 4,000 publications reported in the GWAS catalog to date (MacArthur et al., 2017).

**FIGURE 1:**
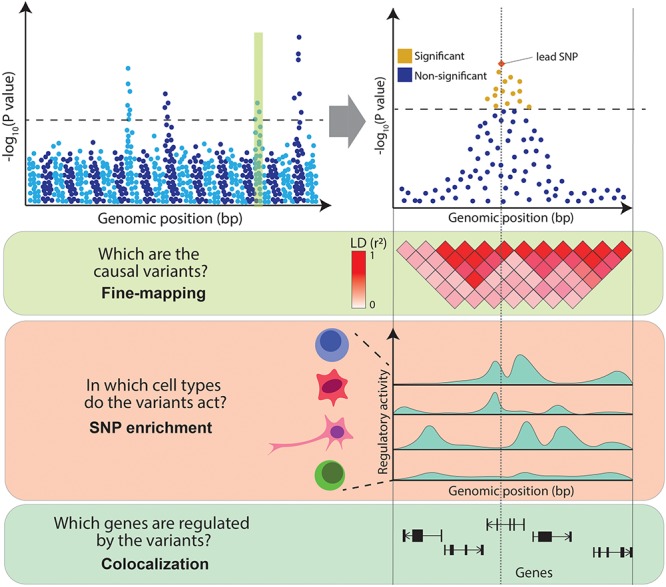
Challenges in interpreting GWAS associations. From the top: Manhattan plot illustrates the association between genetic variants and a trait (e.g., a disease) at a genome-wide level (left panel) and within an example locus (right panel). Variants above the dotted line represent genome-wide significant associations. The panels below illustrate the main challenges in interpreting GWAS associations: high LD between variants (encoded in shades of red), variable levels of regulatory activity of the genomic regions across cell types (peaks of different heights represent different levels of activity of chromatin marks) and multiple genes within the associated locus.

Despite the success of GWAS, the clinical insights derived from their results have been limited. This is due to the difficulty of interpreting GWAS associations. Firstly, neighboring genetic variants are often correlated with one another, as they tend to be inherited together due to co-segregation during meiotic recombination, a phenomenon referred to as linkage disequilibrium (LD) [for a more detailed discussion of LD, refer to the review by Slatkin (2008)]. LD results in multiple variants in a locus being present in the same individual purely due to this correlation. This makes it difficult to distinguish the causal variants underpinning the association. Secondly, it is unclear which cell types are causal to the disease, as the pathophysiology of complex diseases often implicates interactions of multiple cell types. For example, the development of atherosclerotic plaques involves monocytes, lymphocytes, mast cells, neutrophils and smooth muscle (Insull, 2009). It is unclear which cell types are the true drivers of a disease (i.e., in which cell type GWAS variants act) and which are the consequence of the disease pathogenic processes. Finally, over 90% of GWAS variants fall in non-coding regions of the genome and thus do not directly affect the coding sequence of a gene. The accumulation of these variants in DNA regulatory elements (Maurano et al., 2012) and the observation that they can disrupt binding sites for transcription factors (TFs) (Musunuru et al., 2010) suggests that these variants act by regulating the expression levels of genes. However, disease-associated loci often contain multiple genes, making it challenging to distinguish the affected ones. In summary, follow-up studies are necessary to interpret GWAS results and to infer the exact disease-causal variants, the genes they regulate and the cell types in which they act (Figure 1).

Statistical methods designed to tackle these challenges integrate GWAS results with functional genomics data such as gene expression or chromatin activity profiles assayed across a range of cell types and tissues. In particular, fine-mapping aims to define causal variants, SNP enrichment methods prioritize disease relevant cell types and colocalization nominates likely target genes (Figure 1). Here, we review a selection of methods that facilitate translation of GWAS results, focusing on SNP enrichment and colocalization approaches, and we highlight some biological conclusions derived from these studies. We also discuss transcriptome-wide association studies which directly associate genes with diseases. For a detailed analysis of fine-mapping methods, we refer the reader to a previous review (Schaid et al., 2018). Finally, we reflect on some of the challenges and opportunities of post-GWAS research, such as the availability of high-throughput single-cell sequencing platforms, the identification of relevant intermediate phenotypes, the development of polygenic risk scores (PRS), and the systematic application of genetic engineering for GWAS validation.

## Identifying Cell Types Relevant to Complex Diseases

The variants mapped through GWAS provide a strong genetic anchor to complex disease biology and therefore to the development of new therapies. However, going from genetics to function requires robust model systems in which disease-causal cells and tissues can be probed and manipulated. For example, tumor-derived human cell lines have been relevant for the systematic identification of novel drug targets in cancer (Behan et al., 2019). Such model systems provide valuable clues for drug target validation, as they enable us to elucidate the molecular mechanisms of disease, to identify relevant genes and to screen compounds with therapeutic potential at high-throughput. However, for many complex diseases, it is unclear which cells are causal. For instance, independent studies have proposed that rheumatoid arthritis is caused by cells as diverse as T cells (Cope et al., 2007), B cells (Bugatti et al., 2014), macrophages (Udalova et al., 2016), and synoviocytes (Beatrix Bartok, 2010). Psychiatric traits, which involve dysregulation of the central nervous system, pose a similar challenge due to the complex histological structure of the brain. For example, over 20 different cellular models have been used to study bipolar disorder (Viswanath et al., 2015). The lack of ground truth causal cell types makes the functional validation of GWAS variants challenging, as dozens of tissues could be involved in the development of a trait. Statistical methods that integrate GWAS variants either with transcriptome or chromatin annotations assayed across a range of different tissues can help nominate the most disease-relevant cell types.

### Snp Enrichment Analysis Based on Genome-Wide Significant Gwas Variants

Identification of disease-relevant cell types assumes that GWAS variants are overrepresented in genomic regions specifically active in the pathogenic cell types (SNP enrichment). SNP enrichment methods integrate GWAS results with different genomic annotations and prioritize the cell types in which associated variants overlap annotations more frequently than expected by chance. For example, cell type specific activity of a genomic region (e.g., a GWAS locus) can be defined by the expression levels of genes within the region. An approach proposed by Hu et al. (2011) (*SNPsea*) defines as highly cell type specific those genes with high expression in individual cell types as compared to all other cell types. If, for a given trait, GWAS loci are overrepresented (enriched) for genes specifically expressed in a given cell type, that cell type is prioritized. The statistical significance is derived from a permutation-based test in which disease-associated loci are compared with random loci of similar properties (e.g., distance to TSS and gene density) (Slowikowski et al., 2014). The authors used this approach for three different immune-mediated diseases (Crohn’s disease, systemic lupus erythematosus and rheumatoid arthritis), testing for enrichment in gene expression across 79 human and 223 mouse tissues. While lupus-associated variants were enriched in genes specifically expressed in B cells, rheumatoid arthritis variants were enriched in genes specific to CD4+ memory T cells (Hu et al., 2011). This demonstrated that SNP enrichment is a valid approach for cell type prioritization and suggested that variants associated with immune-mediated diseases result in dysfunction of the adaptive immune system.

However, gene expression-based methods use an arbitrary definition of which genes contribute to the SNP enrichment score at each locus and either select a single gene with the highest cell type specific gene expression or include all the genes within the locus (Hu et al., 2011). The caveat of this is that the first approach can select the wrong gene and does not account for the effects of multiple causal genes, while the second approach can dilute the signal by including many genes which are likely not relevant to the tested trait.

**FIGURE 2:**
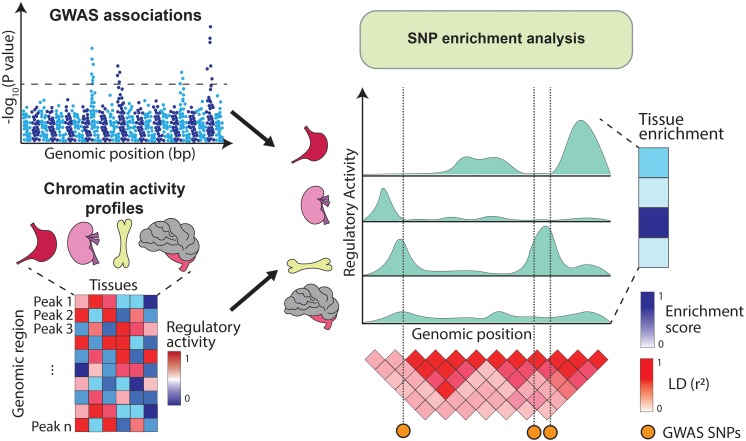
Overview of SNP enrichment analysis using chromatin annotations. SNP enrichment analysis integrates association signals from GWAS (Manhattan plot on the top left) with functional genomics data such as chromatin annotations (heatmap on the bottom left). GWAS SNPs are overlapped with regulatory elements (right panel) and if in a given tissue the overlap occurs more frequently than expected by chance, the tissue is assigned a high enrichment score.

Alternatively, GWAS variants can be integrated with chromatin annotations such as open chromatin regions (assayed by DNase-hypersensitivity or ATAC-seq) (Boyle et al., 2008; Buenrostro et al., 2013), histone modifications (e.g., H3K4me1, H3K4me3, H3K27ac, and H3K27me3) (Bannister and Kouzarides, 2011) or DNA methylation (Frommer et al., 1992). These annotations are profiled using sequencing-based approaches which identify genomic elements with high levels of regulatory activity (i.e., peaks). For example, DNA accessibility peaks indicate regions available for transcription factor (TF) binding, H3K4me3 peaks highlight gene promoters (Barski et al., 2007) and H3K27ac peaks mark active enhancer and promoter regions (Creyghton et al., 2010). As opposed to gene expression, chromatin marks can be physically overlapped with GWAS variants and therefore enrichment analysis can be estimated directly from the SNPs located within the annotations (Figure 2). Initiatives like the Encyclopedia of DNA Elements (ENCODE) (ENCODE Project Consortium, 2012), Roadmap Epigenomics (Roadmap Epigenomics Consortium et al., 2015), and the BLUEPRINT project (Chen et al., 2016) have profiled tens of epigenetic marks across dozens of human tissues, providing rich resources for these type of SNP enrichment analyses.

An early example of SNP enrichment analysis with chromatin annotations overlapped GWAS variants for 447 traits with DNase-hypersensitive (DHS) regions from 348 tissues (Maurano et al., 2012). Using a simple binomial test, this study found that GWAS SNPs were enriched in DHS regions compared to a background set of common SNPs from the HapMap project (International HapMap Consortium, 2003). These SNP enrichment results were tissue-specific, for example, variants for coronary heart disease and body mass index were enriched in DHS regions active in fetal cells. Conversely, variants associated with age-related diseases (e.g., cancer and immune-mediated diseases) were significantly depleted from fetal DHS regions. These findings suggest that GWAS variants could modify the regulatory activity of non-coding elements in a cell-type specific manner.

However, GWAS loci reside in regions of high gene density, which also include higher density of chromatin regulatory elements, which can confound enrichment estimates if not accounted for. To address this issue, enrichment of disease variants in DHS regions can compare GWAS SNPs to random sets of SNPs with similar properties (i.e., LD, gene density and distance to TSS) in a permutation-based approach (*GREGOR*) (Schmidt et al., 2015). By matching SNPs, this approach is robust to both gene and annotation density. Results from this study confirmed that GWAS SNPs are generally enriched in active regulatory regions compared to random SNPs.

In addition to the binary overlap between SNPs and annotations, SNP enrichment analysis can also take into account other peak properties, such as the position of a variant within a peak and the height of the peak (reflecting the levels of regulatory activity). Moreover, SNP enrichment analysis can be extended to chromatin marks other than DHS. For example, *epiGWAS* tests for the accumulation of GWAS variants in chromatin regions defined using ChIP-seq for histone modifications (Trynka et al., 2013). In this approach, variants within each GWAS locus are scored for their distance to the summit of the nearest peak and for the height of the peak i.e., the height (h) to distance (d) ratio (h/d). The contribution to the final enrichment score is determined by a single variant per locus with the highest h/d score, and statistical significance of the enrichment is inferred by comparison to a matched set of random SNPs sampled from the GWAS catalog (MacArthur et al., 2017). This approach is suitable for narrow histone marks, where peak summits can be reliably defined. The authors confirmed that variants associated with LDL cholesterol levels were enriched in gene promoters active in the liver, and that type 2 diabetes variants were enriched in gene promoters active in both liver cells and pancreatic islets. In both cases, the tissues are well understood to play a role in disease biology. The authors also used this approach across immune-mediated traits where pathogenic cell types are less well characterized. This revealed an enrichment of rheumatoid arthritis and type 1 diabetes variants in CD4+ T cell subsets, particularly in regulatory T cells.

One limitation of the above methods, which all rely on random sampling of SNPs to derive a null distribution, is that they make assumptions on the SNP parameters that need to be controlled for in random sampling (e.g., proximity to transcription start site, minor allele frequency, gene density, etc.). However, the presence of hidden confounders could bias the enrichment statistics if uncontrolled for. For example, high LD in a given genomic region can result in inflated SNP enrichment estimates (Trynka et al., 2015). One approach to address this, the *GoShifter* method (Trynka et al., 2015), derives statistical significance by shifting the location of functional annotations within the tested regions while preserving the distance between them. The result is a null distribution of SNP-annotation overlaps due to chance. This approach maintains the local genomic architecture, including the number of tested SNPs in LD, the number of annotations and the distance between the features, therefore controlling for hidden confounders. *GoShifter* confirmed a significant enrichment of rheumatoid arthritis variants in promoter regions specific to CD4+ memory T cells and also detected an enrichment of breast cancer variants in human mammary epithelial cells (Trynka et al., 2015). Both of these cell types are known to be involved in disease.

Given a well powered GWAS, SNP enrichment analysis can provide important insights into disease pathogenic tissues from leveraging the genetic signals. For example, Onengut-Gumuscu et al. (2015) asked if credible sets of type 1 diabetes SNPs (defined with a Bayesian fine-mapping approach) were enriched in functional annotations from the ENCODE (ENCODE Project Consortium, 2012) and Roadmap (Roadmap Epigenomics Consortium et al., 2015) projects (Onengut-Gumuscu et al., 2015). They did so by comparing the proportion of disease-associated SNPs and non-disease SNPs which overlapped functional elements, stratifying variants by their minor allele frequency. Interestingly, type 1 diabetes credible sets were strongly enriched in immune cell enhancers, particularly enhancers active in CD4+ and CD8+ T cells. Conversely, there was no detectable enrichment in enhancers active in pancreatic islets, in agreement with type 1 diabetes being an immune-mediated pathology. In contrast, a separate study profiled open chromatin, TF binding and gene expression in human pancreatic islets and integrated these profiles with GWAS loci for type 2 diabetes and fasting glycemia (Pasquali et al., 2014). The authors used a permutation-based test to estimate enrichments and concluded that glycemia and type 2 diabetes SNPs were strongly enriched in pancreatic islet enhancers, where they disrupted DNA binding by key islet TFs. This illustrates how SNP enrichment can distinguish different disease etiologies based solely on genetic associations, despite the traits sharing similar physiological manifestations.

Once the disease-relevant cell types are identified, subsequent experiments can be carried out to further refine the observed enrichments to the most relevant cell states. For example, we recently followed up the previously reported enrichment of immune disease variants in naive and memory CD4+ T cells, and macrophages (Hu et al., 2011; Fairfax et al., 2014; Trynka et al., 2013, 2015) by stimulating these cell types in the presence of different cytokine cocktails and profiling chromatin landscape with ATAC-seq and H3K27ac ChIP-seq across 55 cell states (Soskic et al., 2019). We observed that, in closely related cell types, the induction of different cell states results in quantitative changes in ATAC-seq and H3K27ac peaks, rather than in the induction of new cell state specific peaks. The broadly applied SNP enrichment methods, which rely on binary SNP-peak overlaps, failed to distinguish disease SNP enrichment between the different cell states. Therefore, we developed a new method (*CHEERS*) to tease apart enrichments in closely related cell types or cell states (Soskic et al., 2019). *CHEERS* asks whether GWAS variants tend to accumulate in regions with highly cell type-specific regulatory activity. SNPs are first intersected with chromatin elements (e.g., chromatin accessibility or ChIP-seq peaks) and are then assigned a score reflecting cell type specific regulatory activity of the region (i.e., how many sequencing reads exist within that region in one cell type as compared to the other cell types). Because this approach is based on cell type-specificity rather than absolute regulatory activity, it can disentangle enrichment patterns across highly similar cell types. We applied this approach to GWAS variants for 11 diseases, using chromatin annotations from our cytokine-stimulated dataset. Variants associated with different subtypes of inflammatory bowel disease (IBD) were enriched in chromatin elements specifically active in the Th1 cell state. For the remaining immune diseases, the strongest enrichment was in early stages of memory T cell activation. This enrichment pattern is important, as it not only nominates T cells as a relevant cell type, but also begins to explain which specific cellular processes are altered in disease. Additionally, a separate study performed SNP enrichment analysis for nine immune diseases using gene expression and chromatin accessibility profiles of 25 immune cell types in resting and activated states (Calderon et al., 2019). Here too, the strongest enrichment was observed among stimulated T cells.

### Genome-Wide Snp Enrichment Analysis

The approaches described so far leverage the signal from genome wide significant variants as shown in Table 1. However, complex traits result from thousands of risk alleles and the majority of trait-associated variants remain undiscovered (Visscher et al., 2017). Thus, restricting the analysis to genome-wide significant variants could limit statistical power to detect biologically important enrichments. This has motivated the development of a number of methods which use all the common variants to estimate enrichments.

**TABLE 1:** Methods for SNP enrichment analysis.

**Method**	**Publications**	**Hypothesis tested**	**Input data**
SNPsea	Hu et al., 2011; Slowikowski et al., 2014	Accumulation of GWAS variants near genes with high tissue specificity	Gene expression, GWAS index variants
EpiGWAS	Trynka et al., 2013	Accumulation of GWAS variants near highly active regulatory elements	Chromatin marks, GWAS index variants
GREGOR	Schmidt et al., 2015	Accumulation of GWAS variants in regulatory elements	Chromatin marks, GWAS index variants
GoShifter	Trynka et al., 2015	Intersection of GWAS variants with regulatory annotations (based on local-shifting of annotations)	Functional annotations, GWAS index variants
fGWAS	Pickrell, 2014	Higher GWAS effect sizes observed if a loci and a SNP overlap a functional annotation	Functional annotations, GWAS summary statistics
CHEERS	Soskic et al., 2019	Accumulation of GWAS variants in regulatory elements with high tissue specificity	Chromatin marks (quantitative), GWAS index variants
GARFIELD	Iotchkova et al., 2019	Higher GWAS effect sizes observed in variants that overlap regulatory annotations	Chromatin annotations, full GWAS summary statistics
RolyPoly	Calderon et al., 2017	Higher GWAS effect sizes observed near highly expressed genes	Gene expression, full GWAS summary statistics
LDSC	Finucane et al., 2015	Accumulation of heritability in variants overlapping a functional annotation	Chromatin annotations, full GWAS summary statistics
LDSC-SEG	Finucane et al., 2018	Accumulation of heritability near tissue specific genes	Gene expression, full GWAS summary statistics

*Selected approaches and methods for enrichment testing of GWAS SNPs in functional annotations included in this review.*

In a method called *fGWAS*, Pickrell reasoned that if GWAS variants were enriched in a given functional category, then SNPs belonging to that category would be more likely to have an effect on the trait (Pickrell, 2014). Using whole genome variants from imputation (Pasaniuc et al., 2014), he modeled the probability of a locus being associated with a disease as a function of its annotations using a hierarchical Bayesian model. When applied to chromatin regulatory maps from 402 tissues and 18 complex traits, *fGWAS* identified enrichment of HDL-associated variants in enhancers specifically active in the liver. Moreover, variants were generally depleted from repressed chromatin regions across all traits. By integrating functional annotations with GWAS statistics, *fGWAS* can also “re-weigh” and discover association signals for variants which did not originally reach genome-wide significance (Pickrell, 2014). An example is the SNP rs6659176, upweighted by *fGWAS* and confirmed to be associated with HDL through an independent study (Global Lipids Genetics Consortium et al., 2013).

In another study, Iotchkova et al. used a logistic regression framework to assess SNP enrichment (*GARFIELD*) and modeled the trait association status of each SNP as a probability (Iotchkova et al., 2019), defined as a function of the variant’s features (i.e., overlap with a functional annotation, distance to the nearest TSS and number of LD proxies). The significant association of a SNP (a binary variable) was tested at several significance thresholds, thus allowing more SNPs to be included in the calculation. The authors applied *GARFIELD* to DHS regions and functional annotations from ENCODE (Ernst and Kellis, 2012) and found that variants associated with height were enriched in DHS elements across all tissues, while ulcerative colitis variants showed tissue-specific enrichment mostly in blood cell types. Interestingly, the authors observed some of the enrichments only at lower significance thresholds. For example, variants associated with beta cell activity index were enriched in pancreatic islets enhancers only at lower significance thresholds (*P* value < 1 × 10^–5^). This suggests that including more trait-associated variants can improve enrichment estimates.

### Enrichment Analysis Based on Snp Heritability

Heritability is the proportion of a trait’s variance that is due to genetic variation. In particular, SNP heritability is the amount of phenotypic variance explained by a given set of SNPs (Yang et al., 2017). A number of methods have been developed to estimate the SNP heritability of a trait using either individual-level genotypes or summary statistics (Yang et al., 2010; Bulik-Sullivan et al., 2015) from GWAS. This gave rise to partitioning heritability approaches, which test for a significant accumulation of trait heritability in different functional categories of the genome. The authors of stratified LD-score regression (*LDSC*) (Finucane et al., 2015) argue that if GWAS variants are enriched in a functional category, then variants falling within that category will explain more trait heritability than other variants. To test for this, Finucane et al., 2015 partitioned all common SNPs into categories based on the functional elements that they overlapped. These categories included 24 unspecific annotations (coding regions, promoters, enhancers, introns, conserved elements and DHSs, among others) as well as histone modification profiles acquired from a variety of cell types. The authors calculated the SNP heritability of variants in each category using GWAS data for 17 traits and defined an enrichment score as the proportion of SNP heritability in a category divided by the proportion of SNPs in that category (Finucane et al., 2015). The authors found that, in general, conserved regions of the genome explained more heritability. Moreover, variants within enhancers specific to disease-relevant cell types also explained a substantial proportion of heritability. For example, liver-specific enhancers were enriched for HDL heritability and enhancers active in the central nervous system captured more SNP heritability of psychiatric traits (e.g., schizophrenia and bipolar disorder) than variants residing in enhancers present in other cell types.

However, one limitation of the *LDSC* method is its dependency on chromatin activity profiles, which are not always available. In contrast, gene expression profiles are available for a far greater number of cell types, including the less abundant ones. LD-score regression applied to specifically expressed genes (*LDSC-SEG*) extends the *LDSC* framework to partition heritability using gene expression profiles (Finucane et al., 2018). If first identifies the top 10% most specific genes expressed in each tissue and extends the regions on each side of the genes by 100 kb. The resulting regions are used as tissue-specific annotations in which variants are partitioned. Because gene expression is available for a wider set of tissues than epigenetic data, this enabled the analysis of less common cell types. The authors used *LDSC-SEG* to integrate expression profiles form GTEx with GWAS data for psychiatric traits and showed evidence of differential heritability enrichment across brain regions. For example, while only cells from the cortex were enriched for schizophrenia SNP heritability, both the cortex and the cerebellum were enriched for bipolar disorder SNP heritability. Subsequent application of *LDSC-SEG* to brain expression data from the PsychENCODE project (The PsychENCODE Consortium et al., 2015) revealed that schizophrenia SNP heritability enrichment was driven by glutamatergic neurons, while bipolar disorder SNP heritability enrichment was driven by GABAergic neurons. Importantly, these psychiatric traits had not been analyzed for SNP enrichment before because of the insufficient number of GWAS-significant variants. This highlights the increased statistical power enabled by including all common variants in the analysis.

Finally, the *RolyPoly* method models the polygenic architecture of complex traits to estimate SNP enrichment (Calderon et al., 2017). In brief, the authors reasoned that variants with higher GWAS effect sizes would tend to be close to genes with higher expression in the causal tissues. Using a regression model, *RolyPoly* estimates the influence of cell type specific gene expression on the variance of GWAS effect sizes in each tissue. The authors applied *RolyPoly* to tissue-specific expression data from GTEx and confirmed a significant enrichment of variants affecting cholesterol levels in genes expressed in the liver and the small intestine. Moreover, they integrated GWAS data with single-cell gene expression profiles from brain tissue (Darmanis et al., 2015) and found a significant enrichment of risk variants for Alzheimer’s disease in genes specific to microglia (Calderon et al., 2017). This agrees with increasing evidence suggesting the immune system is involved in Alzheimer’s pathology (Gosselin et al., 2017).

In summary, SNP enrichment analysis leverages GWAS signals and functional annotations to pinpoint disease-relevant cell types. Multiple approaches have been proposed to estimate enrichment, such as integrating genome-wide significant variants with chromatin or gene expression profiles, as well as partitioning the SNP heritability of a trait based on the functional annotations of the genome. The increasing availability of expression and chromatin data for more cell types and states is expected to improve the granularity of these enrichment signals. This will allow us to confidently nominate the specific cell types and states causally involved in disease.

## Prioritizing Causal Genes at GWAS Loci

Once the most relevant cell types are identified, the next step is to prioritize genes causally involved in disease. Identification of candidate genes is most straightforward for coding variants, which directly disrupt the structure of a protein. One notable example is a locus containing the *TYK2* gene, as well as several gene members of the ICAM family. Variants at this locus have been associated with a number of immune diseases such as rheumatoid arthritis, ankylosing spondylitis, multiple sclerosis and IBD (Franke et al., 2010; Jostins et al., 2012; International Genetics of Ankylosing Spondylitis Consortium et al., 2013; Okada et al., 2014). Importantly, a number of these SNPs are *TYK2* missense variants. Of three independent signals at this locus, at least one is entirely explained by a single coding SNP which confers disease protection (Diogo et al., 2015). This SNP induces a proline to alanine substitution in the catalytic domain of *TYK2*, a kinase that mediates signal transduction downstream of various cytokine receptors (Dendrou et al., 2016). This substitution significantly impairs cytokine signaling, thus altering the communication between immune cells. Surprisingly, even though this variant protects against more than 10 different autoimmune diseases, complete knock-out of *TYK2* causes severe susceptibility to infections (Kreins et al., 2015). This led to the theory that *TYK2* function constitutes a spectrum, with complete abrogation causing immunodeficiency and augmented function increasing susceptibility to autoimmunity (Dendrou et al., 2016). Thus, a compound able to modulate the kinase activity of *TYK2* could be a successful drug candidate for autoimmune disorders.

However, 90% of the variants identified by GWAS are non-coding (Farh et al., 2015) and cannot be easily linked to a candidate causal gene. In contrast, these variants are thought to regulate gene expression via mechanisms such as modification of promoter and enhancer activity or disruption of binding sites for TFs. An example is the 1q13 locus, which contains a variant significantly associated with LDL cholesterol levels and myocardial infarction (Myocardial Infarction Genetics Consortium et al., 2009; Teslovich et al., 2010). This variant was shown to create a new TF binding site, which in turn causes the recruitment of an enhancer-binding protein, sharply increasing the expression of the nearby gene *SORT1* (Musunuru et al., 2010), a regulator of lipoprotein levels in plasma. *SORT1* in turn downregulates the levels of LDL. This makes *SORT1* an interesting drug target in myocardial infarction.

Most disease-associated variants are thought to act by mechanisms analogous to those at the *SORT1* locus. However, GWAS loci often contain multiple genes and identifying the causal genes is challenging. Profiling molecular traits (e.g., gene expression, DNA methylation, TF binding) and integrating them with GWAS results can be useful in linking non-coding variants to their target genes and unveiling the underlying regulatory events.

### Colocalization Analysis

**FIGURE 3:**
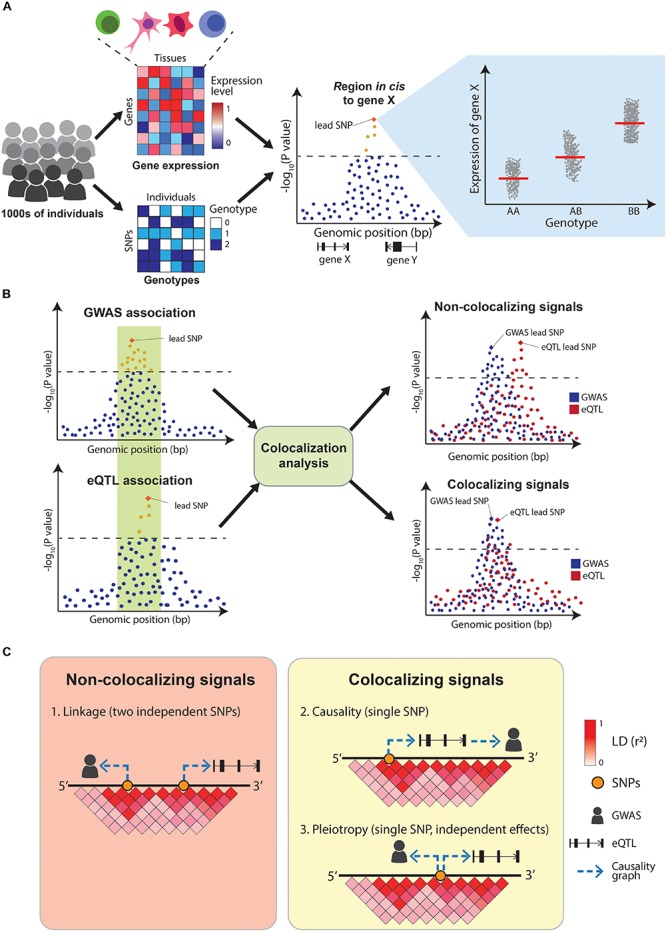
Overview of eQTL-mapping and colocalization. **(A)** In eQTL-mapping gene expression is profiled in thousands of individuals and the expression level of each gene is tested for association with genotypes at nearby (*cis*) SNPs. **(B)** Colocalization compares the association patterns of GWAS and eQTLs at a locus to find if both signals are driven by the same causal variants. **(C)** GWAS and eQTL signals can overlap for three reasons: two independent causal variants in LD (linkage), a single causal variant affecting the GWAS trait via gene expression modulation (causality) or a single causal variant affecting both traits independently (pleiotropy). A positive colocalization supports causality or pleiotropy in favor of linkage.

The quantification of molecular traits such as gene expression across thousands of individuals with different genotypes enables the association of genetic variants with intermediate traits (quantitative trait loci mapping, QTL) (Figure 3A and Table 2). The decreasing costs of high-throughput sequencing have resulted in dozens of QTL-mapping studies, profiling traits as diverse as gene expression (eQTLs) (Nica and Dermitzakis, 2013), protein expression (pQTLs) (Melzer et al., 2008; Yao et al., 2018), exon splicing (sQTLs) (Monlong et al., 2014; Ongen and Dermitzakis, 2015; Li et al., 2018), DNA methylation (mQTLs) (Banovich et al., 2014; Hannon et al., 2016), chromatin acetylation (acQTLs) (Sun et al., 2016; Pelikan et al., 2018), and chromatin accessibility (caQTLs) (Degner et al., 2012; Kumasaka et al., 2016). Of these, eQTLs are the most common, partly because of the robustness of RNA-sequencing technologies. One of the most comprehensive eQTL resources is the Genotype-Tissue expression project (GTEx), which profiled 53 tissues across nearly 1,000 individuals (GTEx Consortium, 2013; Melé et al., 2015). Another initiative, the BLUEPRINT project, measured the transcriptome, together with DNA methylation and histone modifications, in the most abundant cell types in peripheral blood from 197 individuals (Chen et al., 2016).

**TABLE 2:** Methods for colocalization analysis.

**Method**	**Publication**	**Approach**	**Input data**
Regulatory trait concordance (RTC)	Nica et al., 2010	Conditional regression	Individual genotypes
Proportionality test	Wallace et al., 2012	Test for concordance of effects	Individual genotypes
Sherlock	He et al., 2013	Genome-wide comparison of association “signatures”	Summary statistics
COLOC	Giambartolomei et al., 2014	Bayesian test	Summary statistics
gwas-pw	Pickrell et al., 2016	Bayesian test	Summary statistics
eCAVIAR	Hormozdiari et al., 2016	Bayesian fine-mapping and colocalization	Summary statistics
enloc	Wen et al., 2017	Bayesian test for enrichment, fine-mapping and colocalization	Summary statistics
MOLOC	Giambartolomei et al., 2018	Bayesian test for multiple traits	Summary statistics

*Selected approaches and methods used to test for colocalization between GWAS and QTL signals included in this review.*

Integrating QTL maps with GWAS can identify potential molecular mechanisms underlying disease associations. Early examples of this simply assessed whether GWAS variants were also significant eQTLs. A study by Nicolae et al. (2010) combined GWAS results with eQTLs from human lymphoblastoid cell lines and concluded that GWAS SNPs are almost twice as likely to be eQTLs than random sets of SNPs. Similarly, a study by Dubois et al. (2010) concluded that 20 out of 38 (52%) risk loci for celiac disease were eQTLs in primary immune cells. However, these early approaches did not sufficiently control for the genetic architecture underlying GWAS and eQTL signals, resulting in high numbers of false positives findings. In particular, linkage disequilibrium between SNPs makes it challenging to identify which variants within a GWAS and a QTL locus are causally driving the associations. Overlapping eQTL and GWAS signals can be explained by three possible scenarios: (1) two independent causal SNPs in LD with each other (linkage), (2) a single-causal SNP which affects the trait by modulating the expression of a gene (causality), or (3) a single-causal SNP with independent effects on trait and gene expression (pleiotropy). Distinguishing between these scenarios is crucial to appropriately interpret GWAS results (Figures 3B,C). Additionally, eQTLs are abundant (Lappalainen et al., 2013) with 48% of common genetic variants estimated to act as eQTLs for at least one gene (Liu B. et al., 2019), making the overlap between GWAS and eQTL signals likely to happen due to chance. This motivated the development of formal statistical tests that estimate the probability of the overlaps between the two signals being due to chance. These methods are called colocalization tests.

A study by Plagnol et al. (2009) focused on a potentially causal relationship between the 12q13 locus, associated with type 1 diabetes, and the nearby gene *RPS26*. The authors reasoned that if the locus in question increased disease susceptibility via regulation of *RPS26* expression, then the effect sizes inferred from the GWAS and the *RPS26* eQTL (i.e., odds ratios and regression coefficients, respectively) should be proportional. In other words, the SNPs with the highest effects on type 1 diabetes would tend to also have the highest effects on *RPS26* expression, and the direction of effects would be consistent. The authors developed a statistical test for this proportionality (QTLmatch) and concluded that there was no evidence for colocalization at the 12q13 locus. Subsequently, Wallace et al. (2012) revisited this approach and implemented a generalized version into a more robust statistical framework.

An alternative approach described by Nica et al. (2010) first identifies loci with potential colocalizations and next regresses from the eQTL effect the most significant GWAS SNP in a locus. The eQTL association is then re-tested using the residuals from regression. To account for LD in the region, the procedure is repeated for all the SNPs in the region and the impact of the top GWAS SNP is compared to that of other variants. In the presence of a true colocalization, the regression coefficient of the top GWAS SNP results in a significantly larger impact than that of any other variant in the region. This process was implemented into a method called *Regulatory-Trait Concordance* (*RTC*).

Despite the usefulness of these approaches, neither of the two formally compares the odds of colocalization versus a null hypothesis. Instead, they are based on the proportionality of effects or the conditional association between two traits, which can be biased by LD and variable selection (Wallace, 2013). This can result in a large proportion of false positives. Additionally, both approaches require individual-level genotype data, which is seldom available. This motivated the development of methods which could be applied to GWAS summary statistics. Giambartolomei et al. (2014) proposed a colocalization test (*COLOC*) which computes the odds of colocalization compared to the null hypothesis using GWAS summary statistics. The authors identified five mutually exclusive scenarios at any given locus: either (1) the locus is not associated with any of the traits (the null hypothesis, H_0_), (2) the locus is only significant in the GWAS (H_1_), (3) the locus is only a significant eQTL (H_2_), (4) the locus is associated with both traits due to two independent signals (linkage, H_3_) or (5) the locus is associated with both traits due to a single colocalizing SNP (colocalization, H_4_). The probability of each of these scenarios is estimated using a Bayesian framework and any locus where the probability of H_4_ is significantly higher than that of H_3_ (and of any other scenario) is said to colocalize.

Since its release, *COLOC* has become a reference method for colocalization testing. However, a limitation is that it only tests for two traits at a time. Elucidating the full chain of events that connects sequence variation to organismal phenotypes involves more than one molecular trait. For example, a variant can increase DNA methylation, in turn reducing the expression of a nearby gene, impairing cell function and increasing disease risk. Disentangling these effects requires a joint colocalization test for signals from DNA methylation, gene expression and cell function. *MOLOC* expanded the original formulation of *COLOC* to include multiple traits (Giambartolomei et al., 2018). These traits can be independent GWAS, molecular traits or a combination of both. To show the utility of their framework, the authors considered an example case with three traits: GWAS variants for schizophrenia, gene expression and DNA methylation (mQTLs) in the human brain. They showed that adding a third trait significantly increased the power to link variants to genes, as evidenced by 39 new candidate target genes which could only be identified when combining mQTLs and eQTLs. However, these improvements come at the expense of interpretability, increasing the number of possible hypotheses at a locus to 15. Further increases in the number of traits would make the interpretation of colocalization results even more challenging.

Importantly, a trait association signal can result from multiple causal variants (allelic heterogeneity, AH) and recent studies estimate that 20% of the loci identified by GWAS or eQTL-mapping could show AH (Hormozdiari et al., 2017). Methods which assume a single causal variant could potentially misclassify AH cases as colocalizations (Giambartolomei et al., 2014). One method that accounts for multiple causal SNPs per locus is *eCAVIAR* (Hormozdiari et al., 2016) a modified version of the Bayesian method *CAVIAR*, originally designed to perform statistical fine-mapping (Hormozdiari et al., 2014) by estimating the posterior probability of causality for each variant at a GWAS locus (Schaid et al., 2018). Hormozdiari et al. (2016) proposed that fine-mapping could be applied independently to GWAS and QTL associations, and then integrated. Specifically, they defined the probability of a colocalization as the product of the probabilities that the variant was causal in the GWAS and in the eQTL (i.e., the product of the posterior probabilities derived from fine-mapping). Because this approach estimates a posterior probability for each SNP, it does not assume a single causal variant per locus. Instead, *eCAVIAR* can be extended to find colocalizations under the assumption of any number of causal SNPs while accounting for LD.

Colocalization can also be combined with SNP-enrichment, as demonstrated by the statistical method *ENLOC* (Wen et al., 2017). In brief, the authors reasoned that if the majority of GWAS SNPs for a trait are also eQTLs in a given cell type (i.e., if GWAS SNPs are enriched in eQTLs), then most overlaps between the two traits will be driven by true colocalizations. In contrast, if GWAS SNPs are not enriched in eQTLs in that cell type, more of the overlaps are expected to be due to chance. Thus, the authors first estimate an SNP enrichment score and then weigh the priors of their Bayesian model by the identified scores. The authors argue that this approach significantly improves the performance of both fine-mapping and colocalization.

Finally, the effects of GWAS variants are not restricted locally to the genes in close proximity and could have more distal effects (*trans* eQTLs). For example, a GWAS variant could affect the expression of a TF, which would result in a cascade of effects on downstream genes. *Trans* eQTLs are located far away from their target genes and tend to have small effect sizes, which makes them extremely challenging to map at moderate sample sizes due to the burden imposed by multiple testing. In addition, *trans* eQTLs are estimated to be substantially more numerous than *cis* eQTLs (Liu X. et al., 2019), potentially leading to many false positive colocalizations. However, He et al. (2013) reasoned that, while a colocalization between one *trans* eQTL and one GWAS SNP is very likely to be a false positive, the presence of colocalizations between multiple *trans* eQTLs for the same gene and multiple SNPs from the same GWAS is unlikely to be due to chance. Thus, they proposed that the association signals for two traits (e.g., a complex trait and the expression of a gene) could be compared not locally but genome-wide, analogously to comparing two “fingerprints” or “signatures.” If two traits tend to have the same signature, they are said to colocalize. The authors applied their method (*Sherlock*) to integrate summary statistics from a GWAS for type 2 diabetes (T2D) with 3,210 *cis* and 242 *trans* eQTLs specific to the liver (Schadt et al., 2008). This analysis identified four candidate genes regulated by T2D variants, two of which acted *in trans* and would have thus been missed by traditional colocalization approaches. Importantly, three of these four genes (*TSPAN8*, *GNB5*, and *JAZF1*) were supported by previous functional studies. The increasing sample sizes of gene expression studies are allowing us to systematically map *trans* eQTLs (Westra et al., 2013) and will provide more statistical power to detect meaningful colocalizations between GWAS and *trans* eQTLs.

### Application of Colocalization to Complex Diseases

One of the areas where colocalization analysis has been particularly informative is in identifying the mechanisms underlying immune-mediated diseases. A study by Fortune et al. (2015) used colocalization to investigate the shared etiology of complex immune diseases. The authors investigated 126 GWAS loci associated with type 1 diabetes, rheumatoid arthritis, celiac disease and multiple sclerosis and identified 33 to be shared across these four diseases. Colocalization revealed that at 14 of these regions the causal variants were likely to be different. In contrast, the remaining loci showed evidence of a single causal variant affecting all traits. For example, the associations at the *CTLA4* locus colocalized between the three tested diseases. Interestingly, the authors also found three significant colocalizations between type 1 and type 2 diabetes loci, suggesting that these diseases could share certain aspects of their etiology, despite type 1 diabetes having an immune origin.

Colocalization has also pointed to genes and functional elements involved in these diseases. A study by Huang et al. (2017) fine-mapped variants associated with IBD and integrated them with eQTLs mapped in immune cells. The authors found that a large number of IBD variants colocalized with eQTLs in CD4+ T cells (Huang et al., 2017). However, in a separate study immune disease risk variants (including IBD variants) were tested for colocalization with eQTLs across three immune cell types (lymphoblastoid cells, CD4+ T cells and monocytes) (Chun et al., 2017) and it was found that the majority of loci did not colocalize with eQTLs. The authors concluded that GWAS variants could act via more complicated mechanisms and regulate other molecular traits rather than gene expression. A study by Bossini-Castillo et al. (2019) mapped QTLs for gene expression and chromatin traits (histone modifications and chromatin accessibility) in regulatory CD4+ T cells, a rare cell type that plays a central role in regulating the immune response. The authors integrated chromatin and gene expression QTLs with GWAS loci for 14 immune-mediated diseases and identified 253 colocalizations, the majority of which implicated histone acetylation (H3K27ac) QTLs (acQTL). Interestingly, over 70% of these acQTLs were not linked to any eQTL effects, i.e., the loci were associated with local chromatin regulatory activity but not with the expression of nearby genes. A proportion of these colocalizations could represent context-specific eQTLs, which would only be detected upon exposure of the cells to the correct environmental cues. This is known to be the case for other immune cells such as human macrophages, where exposure to cytokines or pathogens has been shown to induce context-specific chromatin accessibility and expression QTLs (Alasoo et al., 2018).

Another area where colocalization has been particularly informative is cardiovascular disease. Franceschini et al. (2018) performed GWAS meta-analyses of two cardiovascular traits (carotid plaque burden and carotid artery thickness) and tested the variants for colocalization with vascular tissue eQTLs, with the aim of investigating the molecular mechanisms underlying cardiac phenotypes. This analysis prioritized two candidate genes (*CCDC71L* and *PRKAR2B*) which colocalized with both traits, suggesting potential disease mechanisms in which regulation of gene expression in arterial smooth muscle impacts artery thickness and plaque formation, ultimately leading to atherosclerosis. In a separate study Liu et al. (2018) integrated GWAS loci for coronary artery disease (CAD) with expression and splicing QTLs mapped in smooth muscle cells from 52 individuals. The authors identified five significant colocalizations (*FES, SMAD3, TCF21, PDGFRA*, and *SIPA1*) and found that increased levels of *TCF21* and *FES* were associated with reduced risk of CAD. Importantly, all of the genes were involved in vascular remodeling, strengthening the hypothesis that gene expression in arterial smooth muscle could have an important impact in local tissue architecture, thus modifying the risk of several correlated cardiovascular traits.

Finally, colocalization analysis can also inform about the relationship between shared genetic architectures across complex traits. A study by Pickrell et al. (2016) used results from 43 GWAS for 42 traits, including neurological phenotypes, anthropometric traits, social traits, immune-mediated disease, metabolic phenotypes, and hematopoietic traits. The authors developed a method (*gwas-pw*) which tested for colocalization between all possible pairwise combinations of these 42 traits and then grouped together those for which there was substantial evidence of colocalization across multiple loci (Pickrell et al., 2016). Most of the traits showed few colocalizations with each other. Nonetheless, the analysis identified two groups of traits (10 traits in total) with a higher number of colocalizations with each other than expected by chance. The first group contained metabolic phenotypes (triglycerides, HDL cholesterol, LDL cholesterol, and CAD), while the second group contained hematopoietic traits (red blood cell volume, hemoglobin concentration and platelet count, among others). The large number of colocalizations in the second group suggests pleiotropic effects across the associated variants, which could indicate that the same variants are able to regulate the differentiation of several independent hematopoietic lineages.

### Twas: Direct Association of Genes and Traits

The examples outlined so far rely on colocalization analyses using genome-wide significant SNPs to nominate causal genes for complex traits. However, the majority of variants contributing to complex phenotypes have not yet been identified, as their effect sizes are too small to be detected at current GWAS sample sizes (Visscher et al., 2017). Another way to gain insights into the biology of complex traits is by directly testing for association between a trait and gene expression (i.e., identifying which genes are expressed at a significantly different level in cases compared to controls in disease-relevant cell types). Given that the number of genes is substantially lower than the number of common variants, using gene expression rather than genotypes for association benefits from a reduced multiple testing burden. Nonetheless, carrying out such a study is currently unfeasible, as it would require profiling gene expression across hundreds of thousands of individuals in both cases and controls, and across dozens of tissues. Alternatively, cell type-specific gene expression profiles can be predicted (i.e., imputed) based on genotypes, thus obviating the need to perform costly RNA-sequencing experiments. *Transcriptome-wide association studies* (TWAS) leverage information from GWAS and eQTL catalogs to predict the transcriptome of cases and controls, thus allowing the direct association of traits and genes without directly profiling gene expression in every individual included in the GWAS (Wainberg et al., 2019).

**FIGURE 4:**
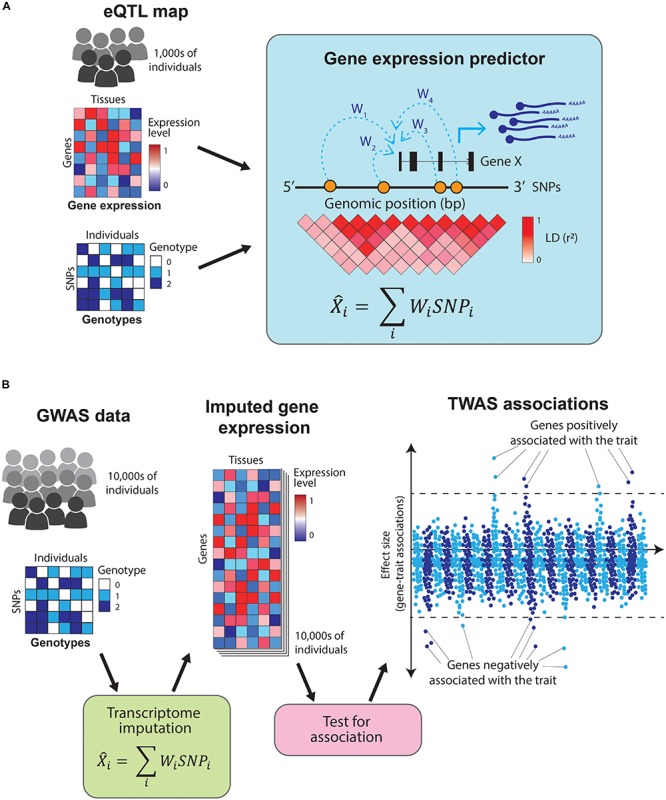
Overview of transcriptome-wide association studies TWAS leverage information from eQTL catalogs and GWAS studies to directly associate traits to genes. **(A)** TWAS use eQTL maps (which contain tissue-specific gene expression and genotypes for thousands of individuals) as a training set to build gene expression predictors. These predictors take the SNPs *in cis* to a gene and estimate its expression levels. **(B)** The resulting predictors are used to impute gene expression values across the hundreds of thousands of individuals in a GWAS study (which contains genotypes but no gene expression data). Finally, the imputed gene expression values are directly tested for association with the GWAS trait, resulting in a set of genes which positively or negatively influence it.

Predicting expression of a gene based on genotypes is possible because gene expression is highly heritable (Wright et al., 2014) and most of the gene expression heritability is attributable to variants in proximity (*in cis*) to the genes (Lloyd-Jones et al., 2017). TWAS uses tissue-specific eQTL maps as reference datasets to train predictors that take an individual’s genotype as an input and estimate their transcriptome levels (Gamazon et al., 2015; Gusev et al., 2016; Figure 4A). These predictors use only information from SNPs *in cis* to the genes and are restricted to genes with highly heritable expression. This prediction process is analogous to genotype imputation and allows for direct association between a trait and the expression of each gene (Figure 4B). Moreover, by focusing on the heritable component of gene expression, it minimizes the confounding by disease-caused changes in gene expression.

*PrediXcan* (Gamazon et al., 2015), an implementation of TWAS, uses an elastic net model to predict gene expression from eQTL catalogs. The authors applied this approach to data from the Wellcome Trust Case Control Consortium (WTCCC) (Wellcome Trust Case Control Consortium, 2007) and identified 41 genes associated with five complex diseases. The majority of these genes were known candidates from GWAS, while others (e.g., *KCNN4* and *PTPRE*) had not been implicated in the diseases before. Importantly, because TWAS directly associates traits to genes, the associations have a clear directionality of effects. As an illustration, a SNP nearby *ERBB3* had been previously associated with type 1 diabetes (Hakonarson et al., 2008). *PrediXcan* confirmed the association between *ERBB3* and type 1 diabetes and found that low *ERBB3* expression increased disease risk (Gamazon et al., 2015). Defining the directionality of effects of GWAS variants, and particularly identifying risk variants which increase gene expression, can nominate effective drug targets and accelerate the development of new therapies.

To overcome the requirement for individual-level genotypes, the authors of *PrediXcan* subsequently derived a mathematical formulation (*S-PrediXcan*) which achieves comparable results using GWAS summary statistics (Barbeira et al., 2018). The authors applied *S-PrediXcan* to over 100 phenotypes across 44 GTEx tissues and found that most of the associations detected were tissue-specific, highlighting the need to profile gene expression in disease-relevant cell types. For example, LDL levels were positively associated with *SORT1* expression only in the liver and negatively associated with *PCSK9* only in tibial nerve. In contrast, schizophrenia was negatively associated with *C4A* expression across 42 of the 44 tissues tested (Barbeira et al., 2018).

Because most of the SNPs used to predict gene expression in TWAS are enriched in regulatory DNA (Trynka and Raychaudhuri, 2013), including epigenetic annotations in the model can improve transcriptome imputation. *EpiXcan* is an implementation of *PrediXcan* which takes into account annotations such as DNA methylation or histone modifications (Zhang et al., 2019). The contribution of each SNP in the prediction is weighted by its overlap with regulatory elements in a Bayesian hierarchical model. When applied to 58 traits and 14 eQTL data sets, *EpiXcan* increased the number of gene-trait associations by over 18% compared to *PrediXcan*. Most of these associations were tissue-specific. For example, TWAS associations with CAD were only detected in arterial tissue, while schizophrenia associations were specific to the brain (Zhang et al., 2019). Moreover, integrating *EpiXcan* with a catalog of chemical perturbations revealed drug repurposing opportunities. An example is ursolic acid, which can reverse the gene expression changes associated with BMI. This compound is currently under investigation for the treatment of obesity (Kunkel et al., 2012).

Another TWAS approach proposed by Gusev et al. (2016) uses a Bayesian predictor to impute gene expression from genotypes. First, the method determines the weights of the Bayesian predictor based on a reference eQTL catalog. The contributions of each variant to the predictions are proportional to its eQTL effects on each gene. Next, gene expression is imputed directly from the GWAS summary statistics. To do this, the authors first use the summary statistics to impute the GWAS effect sizes of all common variants (Pasaniuc et al., 2014) and then multiply these effect sizes by the Bayesian weight of each variant (determined from the eQTL catalog as previously described). Each variant is then re-weighed by its LD with other variants in the locus. Finally, the contribution of all variants proximal to a gene is combined into a single expression-trait association estimate. The authors used this approach to find genes involved in the regulation of circulating lipid levels (HDL, LDL, total cholesterol, and triglycerides). This analysis nominated 665 lipid-associated genes, of which 66 had not been previously identified by any of the independent GWAS (Gusev et al., 2016). The majority of these novel genes showed additional functional evidence from mouse studies. For example, *FTSJ3* expression correlated with fat mass and glucose-to-insulin ratio in mice, while *ITIH4* correlated with LDL levels.

Gusev et al. (2018) subsequently extended their approach to epigenetic data. The authors performed a TWAS to test for association between gene expression in brain tissue and risk for schizophrenia, including as an additional layer of information chromatin marks (i.e., H3K27ac, H3K4me1, and H3K4me3) assayed in 76 lymphoblastoid cell lines. This allowed them to nominate both genes and regulatory elements involved in disease. For example, the authors found two chromatin elements associated with *MAPK3* expression, which was in turn associated with schizophrenia risk. They then functionally validated this association, showing that *MAPK3* is involved in a neuro-proliferation phenotype in zebrafish (Gusev et al., 2018).

Finally, *summary data-based Mendelian randomization* (*SMR*) uses a Mendelian randomization (MR) framework to perform a TWAS analysis (Zhu et al., 2016). MR takes advantage of the fact that an individual’s genotype is independent of confounding factors such as nurture or environmental covariates. In traditional MR, genotypes are used as an instrumental variable to infer causal relationships between an exposure (e.g., the levels of a metabolite or protein) and a trait (e.g., a disease) (Evans and Davey Smith, 2015). In *SMR*, an analogous approach is used to infer associations between gene expression and a trait. In brief, the authors use genetic variants as instrumental variables and estimate the effect size of a gene in a trait as the ratio of the GWAS effect size to the eQTL effect size of a variant affecting the expression of the gene (Zhu et al., 2016). Traditional TWAS approaches impute gene expression from genotypes and then associate genes to traits. However, because imputation is based on the combined effects of multiple proximal variants, TWAS cannot directly point to the individual variants underlying gene-trait associations. In contrast, *SMR* estimates a separate gene-trait effect size from each individual SNP in a locus, thus making it possible to link variants to genes. By comparing the effect-sizes derived from all the SNPs in a locus, *SMR* is able to identify cases in which a single variant affects both gene expression and a complex trait. This test (*HEIDI*) is a form of colocalization analysis (Zhu et al., 2016). However, since most gene-trait effects are small due to polygenicity (Boyle et al., 2017), *SMR* requires eQTL catalogs of very large sample size. The authors applied *SMR* to a large peripheral blood eQTL study (5,311 samples) (Westra et al., 2013) and identified 289 genes associated with body-mass index, waist-hip ratio, rheumatoid arthritis and schizophrenia. Of these, 104 genes showed evidence of a single causal variant. An interesting example includes a locus associated with rheumatoid arthritis which contains the genes *TRAF1* and *C5*. Based on its function, *TRAF1* had been prioritized as the most likely target gene. *SMR* confirmed the prioritization of *TRAF1* and provided evidence of a single causal variant in the region (Zhu et al., 2016).

In summary, colocalization and TWAS prioritize the genes causally involved in complex diseases. Colocalization analysis integrates association signals from GWAS and QTLs in a locus by locus basis to identify instances in which both traits share a causal variant. In contrast, TWAS leverages information from eQTL catalogs to impute gene expression values and directly associate genes to traits. The availability of QTL catalogs from a wider variety of cell types, as well as of larger sample sizes, will improve gene prioritization and translate GWAS results to refined sets of disease-causal genes.

## Future Perspectives in Interpreting GWAS Associations

Enrichment and colocalization analyses have prioritized tissues and genes involved in complex diseases. However, these approaches are largely limited by the availability of comprehensive reference functional data sets. For example, enrichment and colocalization mostly rely on gene expression data from bulk tissues. However, gene expression profiles from bulk tissue are dominated by the most abundant cell types and do not capture information about cell composition and cell type frequencies (Trapnell, 2015). Moreover, colocalization methods are purely observational and cannot establish causality. For example, a SNP could affect both a gene and a trait via independent mechanisms (i.e., pleiotropy), and colocalization cannot conclusively distinguish this scenario from a single causal variant. Thus, candidate genes require additional experimental validation to unambiguously establish causality, for example, by integrating GWAS variants with single-cell assays, or validating candidate genes with gene-editing technologies.

### Integration of Gwas With Single-Cell Genomics

Single-cell genomic assays enable quantification of molecular traits at the single-cell level. For example, multiple existing methods allow profiling gene expression (Picelli et al., 2013; Macosko et al., 2015; Kimmerling et al., 2016; Zheng et al., 2017), chromatin accessibility (Buenrostro et al., 2015), and TF occupancy (Rotem et al., 2015; Grosselin et al., 2019) with single-cell resolution. These assays can resolve the cellular composition of complex organs and tissues, and are used to assemble cells into reference tissue atlases (Regev et al., 2017). Moreover, they can order differentiating cells into time-course trajectories that span different stages of differentiation, an approach called pseudotime ordering (Saelens et al., 2019).

The high resolution of single-cell genomic maps makes them a promising resource for SNP enrichment analysis. This is illustrated by a recent GWAS of hematological traits like hematocrit, hemoglobin and blood cell counts (Ulirsch et al., 2019). In this study, the authors integrated fine-mapped GWAS variants with bulk and single-cell chromatin accessibility profiles spanning a large number of hematopoietic and progenitor cell lineages. The authors developed a SNP enrichment test (*g-chromVAR*) which integrates the quantitative levels of chromatin accessibility in each single cell with the posterior probabilities of causality of each variant inferred from fine-mapping. Enrichment estimates varied throughout the differentiation trajectory and concentrated at specific stages of hematopoiesis. For example, variants associated with platelet counts were progressively more enriched as cells differentiated into megakaryocytes, the precursors of platelets. Conversely, enrichment decreased along differentiation toward the lymphoid lineage. With the rapid increase in the number, depth and size of single-cell datasets, more studies like this will soon be possible and applicable to a whole range of complex traits. However, single-cell genomic approaches introduce new challenges to the current statistical methods, such as data size, sparsity, and high dropout rates (Lähnemann et al., 2020). Thus, it will be essential to develop new statistical methods designed to deal with the intricacies of single-cell data.

Single-cell technologies can also expand the current scope of colocalization. Because the throughput of these assays is growing at an unprecedented scale, it is now possible to profile single-cell transcriptomes in large scale populations of individuals, allowing to map single-cell eQTLs (sc-eQTLs). One such study profiled gene expression in 45,000 single-cells isolated from peripheral blood of 45 healthy individuals (van der Wijst et al., 2018) and identified eQTLs with opposite effects in different cell types in blood. For example, rs4804315 increased the expression of *ZNF414* in NK cells but decreased it in T cells. Moreover, the authors also recapitulated two previously reported monocyte eQTLs for the *HLA-DQA1* and *CTSC* genes and showed that they were specific to the classical monocyte subpopulation (van der Wijst et al., 2018). These results would be difficult to obtain from bulk gene expression measurements. This study serves as a proof of concept and shows how single-cell eQTL associations could rapidly become available for integration with GWAS.

An additional advantage of single-cell sequencing is the possibility of ordering cells into time-course trajectories, thus adding a temporal component to the association models used for eQTL-mapping. This permits the identification of eQTLs with different effect sizes at different stages of differentiation (dynamic eQTLs). Two studies mapped dynamic eQTLs during the differentiation of human induced pluripotent stem cells (iPSCs). The first study investigated iPSC differentiation into endoderm (Cuomo et al., 2020). The authors profiled single-cell gene expression at four time points across 125 iPS cell lines and ordered cells into a time-course trajectory spanning distinct cell states. This uncovered 785 dynamic eQTLs. Interestingly, this study was able to map eQTLs with a cell cycle-dependent effect size. The second study focused on cardiomyocyte differentiation and mapped eQTLs at 16 time points across 19 iPS cell lines (Strober et al., 2019). Here, the authors ordered cells in time-course trajectories based on bulk RNA expression profiles and identified modules of genes which increase or decrease along differentiation. Next, they performed eQTL-mapping using a Gaussian model which accounted for the interaction between genotypes and differentiation time. This resulted in the identification of 550 genes with linear and 693 genes with non-linear dynamic eQTL effects. Interestingly, two dynamic eQTLs which regulated the expression of *SCN5A* (a gene altered in dilated cardiomyopathy) were also GWAS variants for QRS and QT interval duration, thus suggesting that dysregulation of gene expression dynamics could have important phenotypic consequences. Until now, colocalization has not been applied to this type of data. However, as the sample sizes of sc-eQTL and dynamic eQTL catalogs grow, they will become an increasingly important resource for identifying subtle changes in gene expression dynamics which lead to disease.

### Integration of Polygenic Risk Scores With Functional Annotations

Genome-wide association studies variants can be used to identify individuals at high risk of disease. This can be achieved by combining hundreds of disease associated-variants carried by an individual into a single score that reflects their overall genetic risk, a polygenic risk score (PRS) (Chatterjee et al., 2016). The integration of PRSs with epidemiological risk factors such as age, sex, smoking status, diet, or family history of disease could improve the stratification of individuals, potentially resulting in more effective clinical interventions (Torkamani et al., 2018). To build a PRS, a subset of variants is selected based on their GWAS association. Next, each variant is assigned a weight, which corresponds to its standardized effect size (i.e., the odds ratio from the GWAS multiplied by the effect direction). Finally, the genetic dosage of each individual variant (i.e., 0, 1, and 2 according to the number of risk alleles carried) is multiplied by its weight, and all loci across the genome are added into a single score. PRSs are often normally distributed and individuals can be grouped by PRS decile, with those in the top deciles being at highest risk (for a detailed discussion refer to the review by Chatterjee et al., 2016).

Polygenic risk scores performance has increased as GWAS studies increased in sample sizes and larger validation cohorts became available, as shown in CAD (Ripatti et al., 2010; Mega et al., 2015; Abraham et al., 2016; Khera et al., 2016) and cancer (Garcia-Closas et al., 2013; Mavaddat et al., 2015; Maas et al., 2016). The availability of large-scale biobanks (Gaziano et al., 2016; Nagai et al., 2017; Bycroft et al., 2018) has enabled unparalleled improvements in this area by linking genetic information with electronic health records for hundreds of thousands of individuals. Two of the largest PRS studies leveraged UK BioBank data to estimate CAD risk using up to 6.6 million SNPs (Abraham et al., 2016; Khera et al., 2018). Khera et al. (2018) demonstrated that individuals at the highest PRS percentiles were at a risk equivalent to that of carrying a monogenic mutation for familial hypercholesterolemia. Another study used 2.1 million SNPs to build an obesity PRS (Khera et al., 2019) and demonstrated that PRSs can stratify individuals before phenotypic differences appear. While the authors observed no differences in birthweight of individuals at different PRS deciles, these became apparent when individuals reached puberty.

Despite these advancements, polygenic scores face severe challenges. Firstly, prediction accuracy remains low. Secondly, PRSs are based on European GWASs and their transferability between populations is low (Martin et al., 2017, 2019). This is alarming, as it could result in misdiagnosis of individuals in underrepresented populations (Manrai et al., 2016). Finally, little is known about the functional mechanisms underlying PRSs. Some of these challenges are now being tackled using functional annotations.

Prediction accuracy is dependent on the SNPs used to build the PRS. In particular, GWAS effect sizes can be confounded by LD (Bulik-Sullivan et al., 2015). To minimize this, SNPs are pruned by LD and thresholded by *P* value, but this can eliminate causal SNPs in LD with each other. To circumvent this, *LDpred* uses a Bayeseian model to shrink the effect sizes of each variant (Vilhjálmsson et al., 2015) based on a prior that models the effect sizes with an LD-informed normal distribution. The PRS constructed in this way outperformed other methods. *LDpred-func* extended *LDpred* by including the overlap between variants and functional elements in the Bayesian prior (Márquez-Luna et al., 2018). By segmenting the genome into coding, conserved, and regulatory elements, *LDpred-func* improved prediction estimates for height. An equivalent method, *AnnoPred*, also uses a Bayesian model to create functionally informed polygenic scores, outperforming traditional PRSs for breast cancer (Hu et al., 2017). A further study leveraged gene co-expression networks in the brain to identify modules of genes with a common regulation (Hari Dass et al., 2019). Based on these modules, the authors identified genes co-expressed with the insulin receptor and used SNPs in proximity to build a PRS that incorporates known disease biology. Nonetheless, using prior knowledge to design PRSs can introduce bias and requires further evaluation.

Functional annotations can also improve transferability of PRS across populations. Despite the LD difference between populations, most causal variants are thought to be shared (Marigorta and Navarro, 2013). Moreover, they often overlap functional annotations which are also shared between populations (Tehranchi et al., 2019). Thus, overlapping GWAS signals with functional annotations (i.e., functional fine-mapping) can increase the chance of including the functional SNPs in a PRS regardless of the population. A recent study leveraged cell type-specific binding of TFs and epigenetic marks in 245 cell types to identify the annotations most enriched for disease heritability. SNPs overlapping these annotations were used to build PRSs for 29 traits (Amariuta et al., 2020). Using the UK and Japan BioBanks, the authors demonstrated that population transferability improved when incorporating functional annotations.

Biobanks can also help in functionally interpreting PRSs. Richardson et al. (2019) used GWAS variants and UK BioBank data to build 162 PRSs spanning traits as varied as anthropometric measurements, cardiovascular traits, and ICD10 codes. They identified traits correlated with each other based on their polygenic scores and used MR to infer causality. Polygenic scores for triglyceride levels, urate levels, LDL, and gout were significantly correlated with each other. MR analysis revealed evidence that elevated triglycerides cause higher urate production, which in turn increases risk of gout. A similar study derived PRSs for blood traits such as hematocrit and cell counts (Xu et al., 2020) and correlated them with disease PRSs. This pinpointed disease-relevant traits, e.g., the PRS for eosinophil counts was highly correlated with the PRS for allergies.

Finally, gene expression is also beginning to be integrated with PRSs. Võsa et al. (2018) mapped *cis* and *trans* eQTLs in a meta-analysis of 31,684 samples from 37 cohorts. They subsequently identified genes affected by dozens of *trans* eQTLs and proposed that such genes could act as hubs where biological processes converge, potentially accumulating a disproportionate amount of genetic risk for complex diseases. These genes are roughly equivalent to the *core genes* proposed by the omnigenic model (Boyle et al., 2017; Liu X. et al., 2019). To identify these hubs, the authors defined quantitative trait scores (QTS) as the associations between the expression of a gene and the PRS of a disease. They mapped 2,658 eQTS genes, including a group of IFN-regulated genes which were correlated with lupus PRS. In the future, increases in the sample sizes of eQTL studies may enable systematic mapping of cell-type specific eQTSs.

### Validation of Gwas Findings Using Gene Editing

Recent years have seen a rapid expansion in the number and efficacy of gene-editing tools. In particular, CRISPR/Cas9 allows the deletion of specific sections of the genome with high accuracy (Wang et al., 2016). CRISPR-based approaches have been used to systematically knock down genes genome-wide, an approach referred to as CRISPR screening (Koike-Yusa et al., 2014). The applications of CRISPR screening are numerous. For example, it can be used to investigate which genes are essential for cancer growth, which in turn provides a platform for drug target identification (Behan et al., 2019).

Coupling CRISPR-editing platforms with informative functional readouts could be a powerful approach to validate GWAS results. For example, a recent study asked which genes are essential for T cell activation by systematically knocking-down all genes in primary human T cells and measuring proliferation upon stimulation (Shifrut et al., 2018). A second study used a similar approach to investigate T helper cell differentiation in mice (Henriksson et al., 2019). These studies are relevant in the context of complex immune diseases, for which GWAS variants are thought to act during T cell activation and differentiation (Calderon et al., 2019; Soskic et al., 2019). Nonetheless, using CRISPR to follow-up candidate genes requires previous knowledge regarding which functional assays are the most disease-relevant. For example, neuronal cell types are thought to be implicated in psychiatric traits (Finucane et al., 2018), but it is not known which specific neuronal functions are compromised in disease, and thus it is uncertain what the best readout for a CRISPR-screen would be. Selecting informative assays may require mapping the genetic architecture of cellular and intermediate traits. A recent study showed that variants which modulate secretion of monocyte cytokines (cytokine-QTLs) tend to be associated with susceptibility to infection (Li et al., 2016). Thus, a CRISPR-screen to validate infection susceptibility genes should probably assess cytokine secretion. Alternatively, single-cell gene expression can also be used as a readout for CRISPR-screens. Due to its high resolution, single-cell sequencing can match the transcriptome of cells with their corresponding guide RNAs. This is the basis of methods like CROP-seq and Perturb-seq (Dixit et al., 2016; Datlinger et al., 2017) that have been used to investigate which genes are essential in processes such as dendritic cell response with single-cell resolution. In the future, high-throughput phenotyping of human cells will be crucial for identifying the best assays to validate candidate GWAS genes.

Gene-editing approaches can also be used to study the non-coding genome. For example, CRISPR-interference (CRISPRi) uses guide RNAs and a defective version of the Cas9 enzyme to prevent regulatory elements from contacting their target genes (Qi et al., 2013). In contrast, CRISPR-activation (CRISPRa) uses a transcriptional activator fused to the Cas9 protein to enhance transcription (Bikard et al., 2013). These tools can be used to map the function of disease-associated regulatory elements. Moreover, deep mutagenesis employs error-prone PCR to randomly mutate all the nucleotides in a regulatory sequence one at a time (McCullum et al., 2010). Mutagenesis is often coupled either with the expression of a reporter gene like luciferase or with a sequencing-based readout. A recent study used deep-mutagenesis followed by sequencing to study the function of each nucleotide in 20 regulatory elements associated with rare and common diseases (Kircher et al., 2019), including the well-known LDL-associated locus near *SORT1* (Musunuru et al., 2010). This enabled the systematic identification of clusters of nucleotides for which mutation significantly alters gene expression. Importantly, these sites often contained known GWAS SNPs and corresponded to TF binding sites, thus suggesting a molecular mechanism for the implicated variants. Another study investigated loci associated with hematological traits using fine-mapping followed by deep mutagenesis (Ulirsch et al., 2016). The authors found strong regulatory effects for 32 variants (corresponding to 23 lead SNPs from GWAS) of which three had a clear molecular mechanism. These approaches could transform our understanding of how genetic variants affect organismal phenotypes.

Ideally, gene-editing should be performed in disease-relevant cell types (for example, in cells prioritized by SNP enrichment). However, current gene-editing approaches are mostly limited to cell lines. The reasons for this are varied. The application of mutagenesis to primary cells is hindered by the large numbers of cells required and the need to keep cells in culture for prolonged periods of time. CRISPR-editing is further limited by the p53-dependent cellular toxicity which accompanies Cas9-induced double-strand breaks (Ihry et al., 2018). Methodological advances such as better systems for Cas9 delivery (DeWitt et al., 2017; Shifrut et al., 2018) will likely overcome some of these limitations. However, further technological development is needed to routinely apply gene-editing as a follow up strategy for GWAS.

## Conclusion

The integration of GWAS associations with cell type-specific functional data has significantly furthered our understanding of how genetic variation leads to disease. On the one hand, SNP enrichment approaches have enabled the prioritization of cell types and tissues based on their disease-relevance. These methods work by testing for the accumulation of variants in regulatory elements specific to a given cell type. They can either be restricted to genome-wide significant variants or estimate enrichments based on the contributions of all common SNPs. On the other hand, colocalization analysis integrates eQTL and GWAS associations to identify the target genes of GWAS loci, leveraging LD information and association patterns. Moreover, TWAS allows the direct association of genes with phenotypes via transcriptome imputation. These approaches are beginning to reveal the tissues and genes affected in complex diseases like autoimmunity, schizophrenia and coronary heart disease. However, they are limited by the resolution of current functional datasets and cannot establish causality. In the future, we anticipate that the integration of GWAS with single-cell data and the validation of candidate genes via gene-editing and cellular phenotyping will help us translate GWAS findings into clinically actionable gene sets.

## Author Contributions

All authors listed have made a substantial, direct and intellectual contribution to the work, and approved it for publication.

## Conflict of Interest

The authors declare that the research was conducted in the absence of any commercial or financial relationships that could be construed as a potential conflict of interest.
